# Self-Perceived Emotional Intelligence Levels in Nursing Students in Times of a Pandemic: Multivariate Representation

**DOI:** 10.3390/ijerph19031811

**Published:** 2022-02-05

**Authors:** Ángel R. Vargas Valencia, María C. Vega-Hernández, Julio C. Aguila Sánchez, Jose A. Vázquez Espinoza, Ángel G. Hilerio López

**Affiliations:** 1 Centre for Applied Multivariate Statistics Research, University of Colima, Colima 28040, Mexico; avargas22@ucol.mx; 2 Faculty of Education, Contemporary University of the Americas, Morelia 58290, Mexico; 3 Department of Statistics, Higher Polytechnic School of Zamora, University of Salamanca, 37008 Salamanca, Spain; mvegahdz@usal.es; 4 Faculty of Anthropological Sciences, Autonomous University of Yucatan, Merida 97305, Mexico; 5 Faculty of Health Science, University of Alicante, 03690 Alicante, Spain; 6 Faculty of Nursing, University of Colima, Colima 28040, Mexico; jvazquez_espinoza@ucol.mx (J.A.V.E.); ahilerito@ucol.mx (Á.G.H.L.)

**Keywords:** emotional intelligence, nursing, school year, pandemic, multivariate statistics

## Abstract

Self-perceived emotional intelligence in healthcare personnel is not just an individual skill but a work tool, which is even more necessary in times of crisis. This article aimed to determine emotional intelligence as perceived by students studying nursing at the University of Colima, Mexico, a year after the start of the COVID-19 pandemic. A cross-sectional survey of an academic year stratified population of 349 students was conducted, using the Trait Meta-Mood Scale-24 instrument. A global descriptive analysis was performed for each school year. Additionally, an ANOVA was performed, and a Multiple Correspondence Analysis was executed. It is essential to highlight the high percentages for emotional attention within the results. However, a large percentage of students required improvement in emotional attention, clarity, and repair. According to their school year, significant differences were observed among student groups within the three emotional intelligence subscales (*p* < 0.05). Second-year students had low levels in the three subscales of emotional intelligence, while fourth-year students had adequate levels. We established that the scores were different depending on the school year, with a significant decrease in second-year students. The implementation of educational programs could aid in the development of emotional skills in students from the health field, especially in times of crisis.

## 1. Introduction

Self-perceived emotional intelligence in healthcare personnel is not just an individual skill but a work tool, which is even more necessary in times of crisis. According to Salovey and Mayer [1], emotional intelligence (EI) is a concept that refers to the capacity of human beings to manage their own emotions, as well as those of others, in order to maintain or achieve high levels of well-being. In more specific terms, EI is defined by Van Rooy and Viswesvaran ([2], p. 72) “as the set of abilities (verbal and nonverbal) which enable a person to generate, recognize, express, understand and evaluate their own emotions and those of others to guide the thoughts and actions that successfully face the demands and pressure of society and the environment.”

Various authors have devoted themselves to the study of EI in university students. For instance, Extremera and Fernández-Berrocal [3] state that EI is related to variables, such as academic stress, depression, or a student’s academic commitment. Research in this area is also relevant for university students in health majors. In this regard, Mohamedi [4] points out the need to promote EI to improve competencies in nursing students.

Pérez et al. [5] found that students in healthcare degree programs experience an educational environment with a high academic load and inadequate educational models, and this leads students to apply a more significant effort to complete their studies successfully. According to Hinrichs et al. [6], these elements are related to academic burnout syndrome. Additionally, mental health pathologies constitute a silent problem in students of health degrees [7]. If this is not addressed in time, Orak et al. [8] assure that student educational and emotional development will suffer negative consequences and adverse effects in their future work performance as healthcare professionals.

Therefore, the health careers curriculum must contain the development of social-emotional skills that allow students to manage academic stress, coupled with the stress of their clinical practices. However, the curriculum should not stop at skills to manage their emotions but also consider the management and care of patients, especially in degrees, such as nursing, where patient care constitutes a primary focus [9]. In correspondence with this, Jerez-Mendoza and Oyarzo-Barría [10] propose that developing skills, such as self-control, enthusiasm, self-motivation, and empathy, is fundamental in nursing degrees.

For this reason, Erbil et al. [11] assure that the inclusion of education regarding EI in school curriculums, not just in health majors, has had positive effects on students as it has helped them to manage their stress levels. This, in turn, has resulted in better interpersonal relationships and higher levels of psychological well-being and academic success. According to López-Fernández [12], this education should be present in academic careers in health sectors. However, despite the positive results of such inclusion, it is not common to find it in nursing [13].

On the other hand, EI impacts student performance and future work performance. On this matter, others [14] refer to the impact of EI education in clinical practice. The authors point out that EI prepares students to establish good relationships with colleagues, patients, and families. To this end, it is necessary to foster the self-perception of EI, which, when applied to the healthcare setting, is the ability to perceive, understand, and regulate one’s own emotions, manage difficult situations better, and make appropriate clinical decisions [7]. Perestelo-Pérez [15] adds that the self-perception of EI is essential to developing an empathic approach to nursing.

At the University of Colima, previous research was conducted with students of health careers (nursing, medicine, and psychology) on the Emotional Quotient (EQ) [16]. Although it is not an EI study per se, it is the only precedent that we found related to our research topic. This study found a significant statistical relationship between the variables of EQ and the semester grade point average of these students [16]. The author concludes that “high EQ values may contribute as a predictor of positive academic performance” ([16], p. 5). However, these results are from a study conducted more than 15 years ago (2006), and since then, no other research on the subject has been found. Nor is there other research on the EI of students from the University of Colima.

In turn, several studies have demonstrated the psychological effects of the pandemic on students [17,18] and healthcare personnel [19,20]. According to a study by Vinueza et al. [19], during the COVID-19 pandemic, more than 90% of medical personnel presented moderate to severe burnout syndrome. As Carballo and Sánchez [20] point out, the stressful situation generated by a health crisis, such as that of COVID-19, demands more significant support for the emotions of healthcare personnel. These studies noted the importance of EI in adapting to crises and high-stress levels [21], such as that faced by healthcare workers as essential workers during a pandemic.

The main objective of this study is to determine the self-perceived emotional intelligence (EI) of students one year after the onset of the COVID-19 pandemic. The present study addresses the following problem: How is the perceived emotional intelligence in future nurses one year after the COVID-19 pandemic considering the school year. The study’s main contributions are how nursing students’ self-perceived emotional intelligence scores vary by school year. Also, this research contributes to greater visibility of the importance of emotion management in university health students, particularly nursing students, in times of health crises.

The following hypotheses are assumed:

**Hypothesis** **1.** *The students’ self-perceived EI one year after the onset of the COVID-19 pandemic varies by school year*.

**Hypothesis** **2.** *The self-perceived EI improves in students in later school years*.

**Hypothesis** **3.** *The students’ self-perceived EI was negatively affected by the COVID-19 pandemic*.

## 2. Materials and Methods

### 2.1. Design

The investigation consisted of a cross-sectional design on a single sample surveyed in January–February of 2021. Stratified sampling was utilized according to the four school years within the university. The questionnaire was sent to students using their email and social networks with the sharing tool within Google Forms. The variables of this research include sociodemographic characteristics, such as gender, age, school year, and self-perceived emotional competencies.

### 2.2. Sample/Participants

The study population included students from the School of Nursing at the University of Colima, Mexico. The participation rate consisted of 75.5% (349), which resulted in a sample of 349 university students. The sample included representation from all four years of the academic program: 23.2% (81) from the first year, 24.6% (86) from the second year, 30.7% (107) from the third year, and 21.5% (75) from the fourth year. Of those surveyed, 79% were women, and 21% were men. The average age of the respondents was 20 years, with an age range between 17 and 33 years.

### 2.3. Data Collection

Students were invited to respond online to the questionnaire. The questionnaire used to meet the research objective was the Trait Meta-Mood Scale-24 (TMMS-24) by Fernández-Berrocal et al. [22]. The scale used consisted of 24 items and three essential dimensions of EI, each with eight items: emotional attention, clarity of feelings, and emotional repair. It is a reduced scale of the original Trait Meta-Mood Scale, which consists of 48 items and assesses the metaknowledge of the emotional states of the respondent. The responses to the items are in the Likert scale format, meaning a five-point scale ranging from 1 (strongly agree) to 5 (strongly disagree). The first subscale of attention to feelings is assessed by the first eight items and referred to the range participants believe they monitor their feelings. The following eight items belonging to the subscale Emotional Clarity assessed how participants believe they perceive their emotions. Finally, the Emotional subscale repair assessed the participants’ belief in regulating or blocking negative moods and prolong positive ones with the final eight items.

### 2.4. Validity and Reliability

In reference to the psychometric properties of the scale, the authors report high internal consistency for each dimension by Cronbach’s alpha: Attention α = 0.90; Clarity α = 0.90; and Repair α = 0.86 [22]. With respect to the different subscales, the internal consistency obtained in this study were: Attention α = 0.87; Clarity α = 0.93; and Repair α = 0.90.

A score can be obtained for each of the subscales by adding the results of each one. Next, the cut-off points were established by the scale’s authors [22] and classified according to the scale’s parameters. These cut-off points vary depending on the gender of the students and can be observed in Table 1.

### 2.5. Ethical Considerations

The ethics committee of the School of Nursing of the University of Colima approved this study. It is essential to mention that the participants in this study were informed about how the data collected would be used; the confidentiality and anonymity of each respondent were ensured, and the participants proceeded to fill out a consent form upon starting the questionnaire.

### 2.6. Data Analysis

A descriptive analysis was performed for each TMMS-24 subscale of the university students. Previously, the Kolmogorov–Smirnov test confirmed the normal distribution of the data by school year but not by gender. The quantitative variables corresponding to the total attention, clarity, and emotional repair scores were expressed as a mean and standard deviation (SD). The bivariate correlation matrix was also calculated using Pearson’s correlation coefficient to determine the connection between the three TMMS-24 subscales.

Subsequently, a descriptive analysis of attention, clarity, and emotional repair was performed in the school years studied (first, second, third, and fourth). A one-way analysis of variance (ANOVA) was completed to analyze the possible differences in the scores obtained in each of the subscales of self-perceived EI among the groups of students according to the school year, as the data met the assumptions of normality and homoscedasticity. If significant differences were found among the groups of university students, post hoc analyses were performed with a Bonferroni adjustment to determine in which groups these differences were found. Moreover, the Mann–Whitney U test was performed to study possible differences by gender in the three subscale scores obtained. The required significance level was 5%.

A descriptive analysis of the students’ levels of attention, clarity, and emotional repair was then presented using percentages and a bar graph to facilitate the interpretation of results. Contingency tables were also used to record and analyze the relationships between the variables and contrast them. In this case, the EI score was obtained in each TMMS-24 subscale by level and school year.

Likewise, a Multiple Correspondence Analysis (MCA) [23] was performed using these tables to provide a multivariant view. MCA is an exploratory, descriptive technique for analyzing double and multiple-entry tables of qualitative variables. Its objective is to summarize a large amount of data in a reduced number of dimensions, with the least possible loss of information. Its graphical representation is an aid to interpretation: the proximity between variables means correlation. It begins with a data matrix represented in a contingency table with *n* rows and *p* columns, from which a cloud of points Rp and Rn are obtained. Quasi-barycentric correlations were acquired and simultaneously plotted on a graph. In this investigation, and as an example for the interpretation of results of this study, the factorial graphs obtained from this analysis compared the levels between the score results while at the same time describing their relationships, but by school year. In this way, it was possible to appreciate the years of study, which presented better or worse scores on the scale with much more clarity.

It is worth mentioning that the previously mentioned analyses were performed using the IBM SPSS Statistics package, Version 26.0 software.

## 3. Results

The results from the descriptive analysis of the EI subscales demonstrate that nursing students present an average emotional attention score of 27.59 and SD = 6.67, an average clarity of feelings score of 26.35 an SD = 7.66, and an average emotional repair score of 27.20 and SD = 7.26. All subscales were highly correlated, observing direct and highly significant relationships (*p* < 0.001). What is striking is the association between clarity and emotional repair (see Table 2).

Table 3 presents the results of the descriptive analysis of the attention, clarity, and emotional repair subscales for each of the school years studied (first, second, third, and fourth). Significant differences were observed in each of the three EI subscales among the groups of students according to their year: F = 2.728, degrees of freedom (d.f.) = 3, *p* = 0.044 in emotional attention; F = 12.308, d.f. = 3, *p* < 0.001 in clarity of feelings; and F = 6.626, d.f.= 3, *p* < 0.001 in emotional repair. Specifically, post hoc tests showed significant differences (*p* < 0.05) in clarity and emotional repair with those in their second year.

This means that the scores measuring attention, clarity, and emotional repair differed according to the nursing students’ school year, with a significant drop in second-year students. For all three EI factors (emotional attention, clarity, and repair), second-year students had average values below the overall mean, while students in the other school years had higher overall values. It is worth noting the exception of third-year students, who obtained a lower average in emotional attention.

No significant differences by gender were observed for total scores of emotional attention, clarity, and repair (*p*-values > 0.05).

### 3.1. Self-Perceived Emotional Intelligence Levels

**Figure 1:**
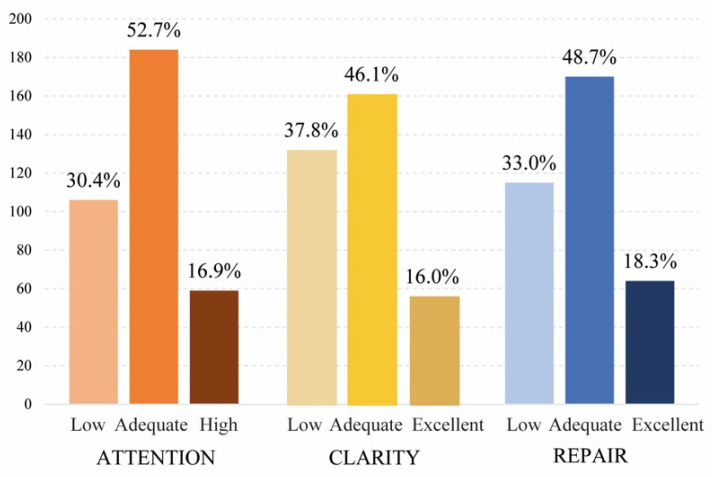
Distribution of students according to EI levels.

The frequency distribution of self-perceived emotional intelligence levels is graphically represented in Figure 1. We observed that more than 30% of the nursing students presented a low level of emotional attention, nearly 40% had low levels of emotional clarity, and 33% showed a low level of emotional repair.

Table 4 shows the distribution of nursing students by year. We observed that slightly over 50% of the students from each year demonstrated an adequate level of emotional attention. The number of students with a low level of emotional attention was smallest in the first year (22.22%) and most significant in the second year, increasing by 17.31%. In successive years, this percentage decreased. Students with excellent emotional clarity were few across all four years. In the second year, only four students were observed to have this level of emotional clarity, and the highest percentage of excellent clarity was presented in the fourth year (22.67%). Comparably, the number of students with excellent emotional repair in the third and fourth years were similar. However, a striking figure was the high percentage in the first year (29.63%) and the low percentage in the second year (11.63%).

No significant association was observed between the genders with levels of emotional clarity and emotional repair (*p*-value = 0.860 and *p*-value = 0.517 respectively), but it was observed with emotional attention (*p*-value = 0.011).

### 3.2. Multiple Correspondence Analysis

Table 5 demonstrates the eigenvalues and inertia indicating the proportion of each dimension of the model’s information. We observed that the first dimension describes more inertia (0.501) than the second (0.363), which was to be expected given that they were obtained through a factorial analysis. This means that the categories present more excellent variance dispersion in dimension 1. Cronbach’s alpha also indicates how the observable variables that constitute the latent variables are correlated.

**Figure 2:**
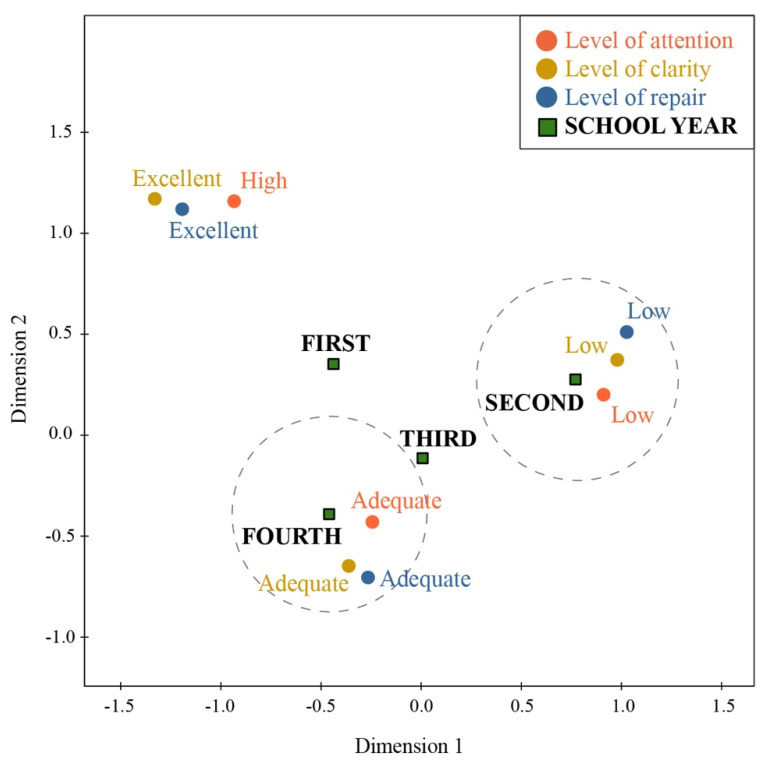
Factorial plane with the joint representation of school years and EI levels.

The factorial graph (Figure 2) shows that excellent levels of clarity and repair were strongly associated with one another and somewhat related to high levels of attention. Similarly, a strong association between adequate levels of clarity and repair were found, which, in turn, though to a lesser degree, appear related to adequate levels in attention. Low clarity levels were also strongly associated with low attention and emotional repair levels. On the other hand, nursing students in their second year showed low levels in all three EI subscales, and those in their fourth year showed adequate levels. Second-year students were associated with adequate or low levels of EI, while first-year students appeared more associated with excellent or high levels.

The differences between EI in first-year students versus second-year students can be explained by the fact that, due to the pandemic, the former was beginning to pursue higher education studies online, so they had to adapt to a different methodology in their training processes and develop self-learning skills. They may have felt remoteness, isolation, or even inevitable abandonment in the educational program, so they had to learn to manage their emotions and recognize, understand, and regulate their feelings themselves. However, the second-year students were facing a slightly more demanding training situation, having to go deeper into the contents being addressed and dealing with the suspension of clinical practices derived from the restrictions in the health units. Therefore, these students may have felt demotivated by being trained in a discipline requiring person-to-person interaction. A lack of clinical experience almost in the middle of their professional training could frustrate students.

## 4. Discussion

As the results indicate, this study allowed us to determine self-perceived student emotional intelligence (EI) one year after the onset of the COVID-19 pandemic. Among the main results, we found high scores for emotional attention. However, 47.3% (30.4% low + 16.9% high) of the students required improvement in attention because excessive attention is just as harmful as too little attention; another 37.8% needed improvement in clarity, and 33.0% required improvement in emotional repair. According to their school year, there were differences within emotional attention, clarity, and repair among student groups. We found that second-year students had low levels in the three subscales of emotional intelligence, while fourth-year students had adequate levels.

The application of the TMMS-24 yielded significant results in nursing students at the University of Colima, Mexico. The first is that more than half of the students presented an adequate level of emotional attention, while 16% had excellent emotional clarity, and 18.3% had excellent emotional repair. These findings underscore the need for 47.3% of the students to improve their emotional attention, while 37.8% could work to obtain adequate levels of emotional clarity, and another 33% need to improve their emotional repair. In this regard, Fragoso-Luzuriaga [24] notes that it is crucial to develop all EI skills because they are interconnected.

The results of this investigation partially agree with those obtained by Estrada et al. [25], in which university students presented a high average on the emotional repair subscale, followed by the clarity subscale.

According to Ramos et al. [26], high emotional intelligence is given by moderate to low emotional attention, coupled with high scores on clarity of feelings and reparation. Accordingly, the students in our study did not generally demonstrate high emotional intelligence; they showed high scores in emotional attention and lower scores in clarity of feelings. In this regard, Gorostiaga et al. [27] add that high rates of emotional attention, such as those found in our study, may imply more significant concern and personal distress on the part of the subjects towards the problems of others.

The present study finds significant differences in students’ emotional intelligence across school years. This disagrees with the study by Arntz and Trunce [28], who observed levels of emotional intelligence among cohorts of students, with no tendency to increase or decrease as the years progressed. In turn, this is not consistent with the idea that samples of university students tend to be more homogeneous [29] than samples of students from earlier levels (secondary school and high school). According to Páez and Castallo [30], this happens because they are part of a small group that passed school and success filters to enter university.

However, our results indicate that second-year students had a lower EI than others. This may be caused by the fact that they were the only students who had exclusively virtual classes since entering university due to the pandemic. In contrast, the fourth-year students showed an adequate EI. This can be explained by the fact that these students had online classes for the last two years and have also had professional internships because students from the most advanced years joined the hospitals in the face of the health emergency. This increased professional practice may have contributed to their improved emotional intelligence.

In our study, we found no significant differences by gender, and this is in agreement with the study results by Estrada [25], who observed adequate management of emotions in both sexes. Similarly, the study by Fernández-Berrocal et al. [22] found no gender differences in any of the three subscales. However, our results contrast with other studies [31,32] that highlight that women express their emotions more quickly than men. According to Sánchez-Núñez et al. [31], this “equality” may be because nowadays, these stereotypes about how men and women handle their emotions are being left behind.

In addition, our results show that students presented high percentages in the low levels of the 3 IE subscales, which need to be improved. Related to this, Liébana-Presa et al. [33] indicate a positive relationship between emotional intelligence and the academic efficacy dimension of burnout. According to this study, high levels of emotional intelligence and emotional awareness in nursing students help them be perceived as more effective in their studies. This result also means that they may have tremendous academic success and professional development. The study by Guo et al. [34] confirms that the lower the EI and self-efficacy of students, the higher the risk of academic procrastination.

Another study [35] showed that the higher the emotional clarity, the higher the personal fulfilment, which is a fundamental aspect in future nurses. Likewise, the higher the perceived ability of students to regulate and understand their emotions, the lower the probability of depression, anxiety, or stress [36]. For this reason, these authors [36] recommend promoting emotional development in school curricula; this would encourage a culture of self-care and care for others and provide them with tools to enrich and protect their mental health.

Corresponding to this, Guiliano and Guiliano [37] concluded in their research that including the emotional aspect in the teaching–learning process is vital for the comprehensive training of nursing students, and this is an indicator of educational quality in the healthcare sector. In addition, this training should be present throughout the entire career, both in teaching and in clinical practice. Moreover, the authors add that it should be done collaboratively and in constant evaluation so that students can measure their evolution [4].

This study provides results from university students in different school years. However, when completing the literature review, no similar research was found about analyzing EI in different academic stages of higher education. Nevertheless, a study [38] showed that the pandemic experienced due to COVID-19 psychologically affected students and, above all, healthcare personnel who had to face complex situations and high levels of stress. Although our results do not confirm that self-perceived student EI was negatively affected by the COVID-19 pandemic, we observed in research before the pandemic that nursing students showed higher EI scores, at least one point above the current ones [14,39].

In the case of Mexico, a recent study [40] showed that nursing education has been more concerned with developing skills, competencies, and aptitudes related to intellectual intelligence than emotional intelligence. Based on this, the authors recommend that nursing education plans include the development of EI skills in their students, considering that their work requires a high emotional demand, for which the university does not prepare them [40].

## 5. Conclusions

The self-perceived EI of students one year after the start of the COVID-19 pandemic presents a higher average score in emotional attention, followed by emotional repair and a lower average score in the clarity of emotions. An adequate level of emotional attention was presented by 52.7% of the nursing students. However, 30.4% showed a low level, and 16.9% show a high level. These results indicate the need to improve EI in those small groups with low levels. It should be noted that while in all courses there was a group of students with high EI, the number of students who could improve their EI was much higher as it was explicitly low or simply adequate. Regarding clarity of feelings, only 16.0% showed an excellent level, similar to what occurred in emotional repair with 18.3%. These scores measuring emotional attention, clarity, and repair were different according to the school years of the nursing students, emphasizing a significant decline in second-year students. 

The multivariate analysis showed that second-year nursing students were associated with low levels in the three EI subscales, while fourth-year nursing students showed relationship with adequate levels. The adequate levels of EI in the second year could be related to the experience of having attended classes in person before the pandemic; as they moved to school and other activities due to social distancing, we assumed that they may be in the process of school adaptation.

The first-year students had better levels of IE, as they were already developing their school activities with technological mediation, which explains why they were in a much better process of adaptation than the second-year students and the theoretical content subjects. Third-year students were associated with adequate or low levels of EI, while those in their first year were not so much related to these levels but were related to excellent/high levels. Thus, the self-perceived EI of students improved with the following school year. The TMMS-24 was a crucial tool to understanding the emotional intelligence of nursing professionals, allowing for improvement in terms of prevention and treatment of patients, considering the results obtained from these professionals.

## 6. Limitations

The strengths of this study include, to a large extent, the sample used, as it involved collecting data in the four school years for the entire School of Nursing. This allowed for a broader view of the self-perceived emotional intelligence of these students throughout their entire process of academic development a little less than a year after the start of the pandemic, making the data of this research incredibly recent (January–February 2021). In addition, this study examined the self-perceived emotional intelligence with multivariate statistics, where the relationship of the four school years and these student levels of emotional intelligence could be observed. This is where the study will serve as a guideline for other colleges and universities that want to determine self-perceived student EI during or after the pandemic or during any moment the students may be experiencing. It also highlighted the importance of self-perceived EI in future health professionals.

The work throughout this investigation also had some limitations. One of these limitations was that the sample used consisted of students from only one university. In the future, it would be beneficial to expand the sample to other universities within the country and even from around the world. Furthermore, as a cross-sectional study, the establishment of casual relationships was not allowed. Future research should be of a longitudinal character which would allow for the analysis of variations in EI over time. It should be considered that the measurements were based on self-reports, which implied the possibility of a social desirability bias. Although the perception of emotional capacities was critical for emotional adjustment, it would be interesting to obtain more objective indicators by studying the concordance between the different methods. In addition, it would be interesting to include qualitative methodology (focus groups) in the study of this topic to have a broader view of the perception of the study subjects.

## Figures and Tables

**Table 1:** Cut-off points according to the gender of the TMMS-24 scale.

Dimensions	Gender	Score	Scale
Attention		≤21	Low attention
	Male	22 to 32	Appropriate attention
		≥33	High attention
		≤24	Low attention
	Female	25 to 35	Appropriate attention
		≥36	High attention
Clarity		≤25	Low clarity
	Male	26 to 35	Appropriate clarity
		≥36	Excellent clarity
		≤23	Low clarity
	Female	24 to 34	Appropriate clarity
		≥35	Excellent clarity
Repair		≤23	Low repair
	Male	24 to 35	Appropriate repair
		≥36	Excellent repair
		≤23	Low repair
	Female	24 to 34	Appropriate repair
		≥35	Excellent repair

**Table 2:** Matrix of correlations between TMMS-24 subscales.

	Attention	Clarity	Repair
Attention	1	0.467 **	0.361 **
Clarity		1	0.670 **
Repair			1

** *p* < 0.01.

**Table 3:** Descriptive analysis per year.

		M	SD	ESM	F	*p*-Value
Attention	First year	28.543	6.630	0.737	2.728	0.044
	Second year	26.326	6.951	0.750		
	Third year	27.018	6.367	0.616		
	Fourth year	28.813	6.557	0.757		
	Total	27.587	6.665	0.357		
Clarity	First year	27.494	7.156	0.795	12.308	<0.001
	Second year	22.593	6.862	0.740		
	Third year	26.430	8.010	0.774		
	Fourth year	29.307	6.942	0.802		
	Total	26.350	7.664	0.410		
Repair	First year	28.864	7.476	0.831	6.626	<0.001
	Second year	24.465	6.844	0.738		
	Third year	27.224	7.329	0.708		
	Fourth year	28.520	6.556	0.757		
	Total	27.203	7.257	0.388		

**Table 4:** Distribution by the level of attention, clarity, and repair by year.

		Attention		Clarity		Repair	
Year	Level	*n*	%	*n*	%	*n*	%
First	Low	18	22.22	26	32.10	21	25.93
	Adequate	**46**	**56.79**	**39**	**48.15**	**36**	**44.44**
	High/Excellent	17	20.99	16	19.75	24	29.63
	Total	81	100.00	81	100.00	81	100.00
Second	Low	34	39.53	**51**	**59.30**	**42**	**48.84**
	Adequate	**39**	**45.35**	31	36.05	34	39.53
	High/Excellent	13	15.12	4	4.65	10	11.63
	Total	86	100.00	86	100.00	86	100.00
Third	Low	35	32.71	37	34.58	36	33.64
	Adequate	**58**	**54.21**	**51**	**47.66**	**53**	**49.53**
	High/Excellent	14	13.08	19	17.76	18	16.82
	Total	107	100.00	107	100.00	107	100.00
Fourth	Low	19	25.33	18	24.00	16	21.33
	Adequate	**41**	**54.67**	**40**	**53.33**	**47**	**62.67**
	High/Excellent	15	20.00	17	22.67	12	16.00
	Total	75	100.00	75	100.00	75	100.00

Note: Higher *n* and % of students for each EI subscale are indicated in bold.

**Table 5:** Explained Variance of the multiple correspondence model.

Dimension	Cronbach’s Alpha	Variance Accounted for		
		Total (Eigenvalue)	Inertia	Variance %
1	0.668	2.005	0.501	50.137
2	0.415	1.452	0.363	36.295
Total		3.457	0.864	
Mean	0.562	1.729	0.432	43.216

Cronbach’s mean alpha is based on the mean eigenvalue.

## Data Availability

The data used to support the findings of this study are available from the corresponding author upon request.
